# Supplementary material for: Retrospective study of prognostic factors in pediatric invasive pneumococcal disease
Source: PeerJ. 2017 Jan 25;5:e2941. doi: 10.7717/peerj.2941 (PMC5270593; doi:10.7717/peerj.2941)
Supplement: Supplemental Information 1 [file peerj-05-2941-s001.pdf]

No.	Site	Sex	birth day	Age	AdmDate	DisDate	Underlying	WBC	Hb	Plt	Seg	CRP
1	P, B	F	1984/8/7	17	2001/1/9	2001/1/17		13080	9.6	233	78.5	16.7
2	B	F	1990/12/9	14	2004/2/17	2004/2/27	T cell NHI	12100	11.2	110	95.1	0.43
3	P	F	1998/2/6	3	2001/1/3	2001/1/27	BA post op	16700	5.2	323	90	29.8
4	P	F	1998/3/21	3	2001/11/23	2001/12/3		22840	11.9	330	84	1.74
5	P	F	1998/7/1	6	2004/9/23	2004/10/6	CHD, TAF	9500	9.4	196	88	11.5
6	P,B	F	2000/8/27	4	2004/5/10	2004/5/25		21400	11	351	83	7.45
7	P,B	M	2000/10/31	3	2003/10/4	2003/10/17	Rectal sten	37900	13.2	356	87	12.1
8	P	F	1999/12/12	1.5	2001/6/2	2001/6/9		14640	9.9	378	72	30.4
9	P	M	1999/12/4	4	2003/10/16	2003/10/31		2300	10.9	271	54	47
10	P, B	M	1998/3/30	4	2001/10/20	2001/10/31		11700	12.5	325	90	1.01
11	P, B	M	2000/6/2	4	2004/10/23	2004/11/3	choledocha	7400	12.9	99	70	5.84
12	P, B	M	1997/3/16	5	2002/3/1	2002/3/20		11600	11.9	263	78	3.83
13	P	F	2001/1/2	1	2002/2/15	2002/2/28		11160	12.5	273	81	30.2
14	P, B	M	2002/5/7	3	2004/9/25	2004/10/6		18400	11.4	480	55	4.93
15	P,B	M	2000/11/8	4	2004/9/29	2004/10/6		20100	11.1	338	80	7.23
16	P	M	1997/11/7	3	2000/12/21	2001/1/4		33630	12.4	390	91	3.39
17	B, OME	M	2000/9/4	4	2004/9/21	2004/10/2		17100	12.9	445	63	7.72
18	P	M	1998/2/3	3.5	2001/8/9	2001/8/9		2670	12.3	58	80	33.4
19	P, B	M	2001/8/9	5	2006/2/2	2006/2/18		16600	13.1	185	80	
20	P	M		6	2002/9/19	2002/9/27	Seizure	29760	11.4	264	75	11.58
21	P, B	M		2	2002/10/2	2002/10/23		5800	11.4	108	78	31.5
22	M	M	2002/5/24	1	2003/4/14	2003/4/15		8100	11.6	487	71	27.4
23	P, B	F	1999/6/14	3	2001/12/1	2001/12/11		17410	12.3	261	62	0.01
24	P	M	2000/8/27	3	2003/1/12	2003/2/17		10120	9	298	77	43
25	P	M	2001/10/3	0.5	2002/4/27	2002/5/3		22840	12.2		61	
26		F	2000/8/11	2	2002/9/17	2002/9/21		3850	9.3	186	63	27.6
26	Pneumonia	F	2000/8/11	2	2002/9/17	2002/9/21		3850	9.3	186	63	27.6
27	Pneumonia	M	1998/6/7	4	2002/10/5	2002/10/30		3000	12.8	2.1	61	35.58
28	P, B	F	1998/8/14	5	2002/10/12	2002/10/26		4240	12.2	179	79	25.2
29	P	F	2000/10/5	2	2003/2/10	2003/3/26		2070	12.9	34	83	49.6

30 P, B	M	1996/11/6	6	2002/11/17	2002/12/1		34620	12.6	348	91	
31	F	2002/9/28	1	2003/12/29	2004/1/12		8900	9.1	254	70	31.5
31 P	F	2002/9/28	1	2003/12/29	2004/1/12		8900	9.1	254	70	31.5
32 P	M	2001/2/8	2	2003/2/9	2003/2/16		17790	11.1	458	80	1.34
33 P, B	F	2000/8/23	4	2004/12/20	2004/12/27		19700	11.9	567	78	2.88
34 P	M	2004/3/31	1	2005/3/23	2005/4/2		27300	9.8	660	86	30.7
35 P, B	M	2004/5/10	1	2005/9/18	2005/9/23		16000	12.3	313	55	7
36	M	2004/2/19	0.5	2004/7/27	2004/8/13		11000	11.3	708	42	4.31
37	F	2002/5/16	1.5	2003/11/20	2003/12/27		3200	6.3	15	38	44.9
36 P, B, M	M	2004/2/19	0.5	2004/7/27	2004/8/13		11000	11.3	708	42	4.31
37 P	F	2002/5/16	1.5	2003/11/20	2003/12/27		3200	6.3	15	38	44.9
38 P, B	F	2004/1/25	1	2004/11/6	2004/11/16		33500	12	545	37	1.87
39 P, B	M	2004/2/3	1.5	2005/8/26	2005/9/12		39600	10.4	957	87	27.5
40 P, B	M	2000/9/27	4	2004/5/30	2004/6/9		4300	10.3	180	76	12.5
41 P, B	M	2000/7/5	4	2004/12/17	2004/12/23		22900	12.8	321	88	16.3
42	M	2000/11/17	5	2005/12/11	2005/12/21		24000	13.6	479	77	0.5
42 P, B	M	2000/11/17	5	2005/12/11	2005/12/21		24000	13.6	479	77	0.5
43 P, B, M	M	2002/1/13	3	2005/7/18	2005/8/7		25500	13.6	438	88	21.3
44 B, M	F	2005/12/4	0.4	2006/4/13	2006/6/20	Subdural e	18400	10.4	602	90	20.5
45 P, B	F	2003/1/30	2	2005/1/20	2005/1/27		18300	12.5	312	62	2.89
46 P, B	M	2000/6/23	4	2005/3/26	2005/4/8		13300	12.3	357	81	1.21
			3.3803922				15632.75	11.22157	327.582	73.05098	17.54479
			3.3803922				15632.75	11.22157	327.582	73.05098	17.54479
47 P, B, E	F	2004/10/25	4	2004/10/25	2004/11/22		2000	12.9	133	12	21
48 P	F	2004/9/29	3	2004/9/29	2004/11/1		6000	11.4	76	54	32
49 P	M	2004/8/10	3	2004/8/10	2004/8/28		29100	10.4	257		27.1
50 P	M	2004/7/27	3	2004/7/27	2004/8/11		16800	10.9	168	86	30.2

Abx	
Keflin+GM	
Targocid	
OX	VA. CTX
AM+Ery	Aq-PCN
CTX	
CXM,rulid	VA
AM	CTX
CXM	
CTX+Aqua	
AM	keflin, Aqua
AM	
AM	CTX
AM, CXM	CTX
AM+GM	Aqua
CXM	
AM,Aqu,C	CTX
Cefmezine	CXM+Aqua
VA+CTX	
CXM+Ery	CTX+VA
OX	Aqua allergy
CXM+Ery	CXM,Aqua
VA+CTX	
Aqua	
CTX	CTX+VA
AM	OX
CTX+VA	
CTX+VA	
VA+CTX	Aqua
CTX+Ery	CTX
VA+CTX	CTX+Aqua

Aqua+CTX	VA+CTX
VA	Aqua
VA	Aqua
Aqua	
CXM	
CXM	
CXM	
AM+GM	VA
CXM	Aqua+CTX
AM+GM	VA
CXM	Aqua+CTX
CXM	Cefamezine
CTX	VA
CXM+Rul	CXM
CXM+Ery	CXM
CTX+Clin	VA+Rulid
CTX+Clin	VA+Rulid
VA+CTX+Ery+acyclovir	
VA+CTX	
CXM	
CXM	CXM+Rulid

PCN+CTX	VA, CTX
CTX	
